# Supplementary material for: CD99Targeted Irinotecan Containing Nanoparticles Show Twenty-Fold Greater Anti-Tumor Effect Than Free Irinotecan For Treatment of Ewing Sarcoma
Source: bioRxiv. 2026 Jan 6:2026.01.06.697855. Preprint. [Version 1] doi: 10.64898/2026.01.06.697855 (PMC12803216; doi:10.64898/2026.01.06.697855)
Supplement: Supplement 1 [file NIHPP2026.01.06.697855v1-supplement-1.pdf]

bioRxiv preprint doi: https://doi.org/10.64898/2026.01.06.697855; this version posted January 6, 2026. The copyright holder for this preprint (which was not certified by peer review) is the author/funder, who has granted bioRxiv a license to display the preprint in perpetuity. It is made available under aCC-BY 4.0 International license.

Supplemental Figures and Legends

bioRxiv preprint doi: https://doi.org/10.64898/2026.01.06.697855; this version posted January 6, 2026. The copyright holder for this preprint (which was not certified by peer review) is the author/funder, who has granted bioRxiv a license to display the preprint in perpetuity. It is made available under aCC-BY 4.0 International license.

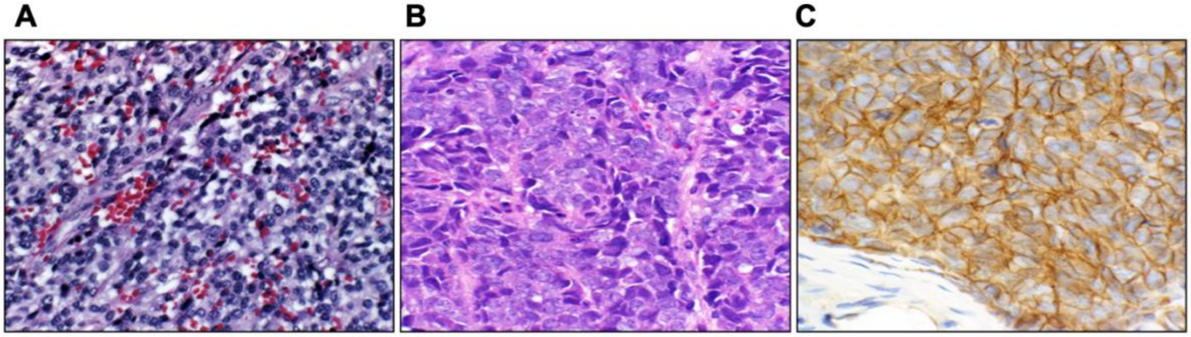

Supplement Figure 1. Establishment of a human Ewing Sarcoma xenograft mouse model. (A) H&E image of a subcutaneous flank tumor generated by implantation of human Ewing sarcoma cells into immunodeficient mice (B) H&E image of primary human Ewing (C) Immunohistochemical staining to show expression of CD99 on the surface of both xenograft and primary tumor cells

A

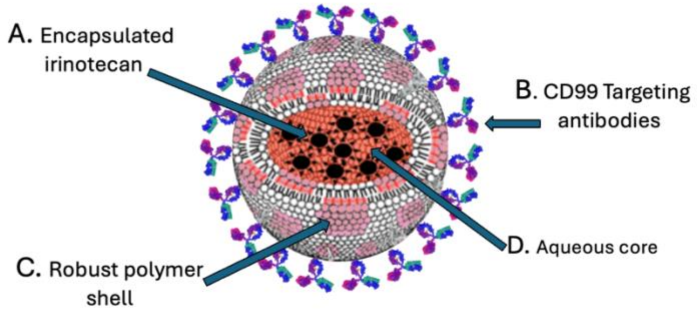

A. Substances encapsulated and deployed in nanoparticles: s*mall molecule cytotoxics (e.g., irinotecan)*
B. CD99 targeting antibodies conjugated to nanoparticles: *human monoclonal anti-CD99*
C. The nanoparticle lipid shells are PEGylated (for immune stealthiness)
D. Use of crosslinked polymeric lipids (i.e., hybrid polymerized liposomal nanoparticles) enhances favorable drug release (DMET and PK/PD) & lysosomal release (bypassing the MDR1 drug resistance mechanism)

B

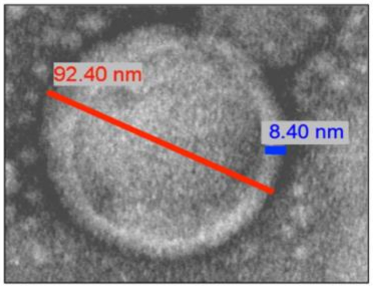

C

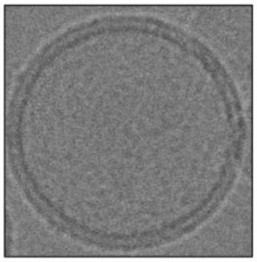

D

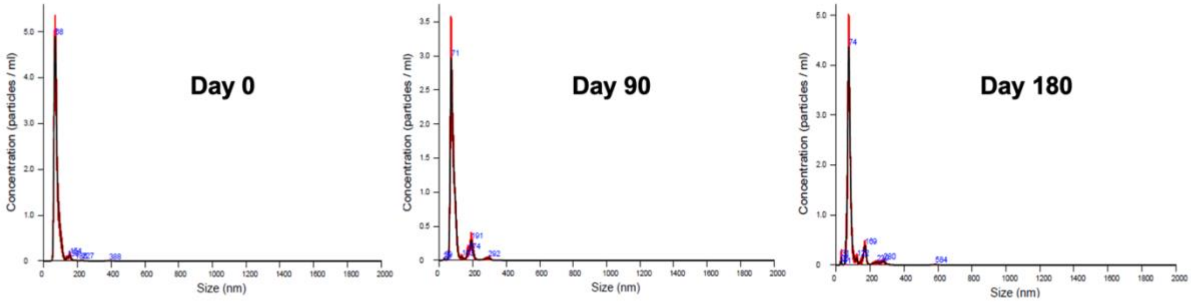

bioRxiv preprint doi: https://doi.org/10.64898/2026.01.06.697855; this version posted January 6, 2026. The copyright holder for this preprint (which was not certified by peer review) is the author/funder, who has granted bioRxiv a license to display the preprint in perpetuity. It is made available under aCC-BY 4.0 International license.

Supplement Figure 2. Schematic and Structural Characterization of Hybrid Polymerized Lipid Nanoparticles (HPLNs). (A) Diagram illustrating the composition of HPLNs, which consisted of cholesterol, hydroxy soy phosphatidylcholine (PC), pegylated distearoylphosphatidylethanolamine (DSPE), and pegylated 10,12-pentacosadiynoic acid (PCDA). Antibodies are conjugated to maleimide-terminated PEGylated DSPE micelles via reduced sulfhydryl bonds to form covalent linkages. (B) Transmission electron microscopy (TEM) image show the spherical morphology of nanoparticles, approximately 90 nm in diameter. (C) Cryo EM image shows the hollow aqueous core of nanoparticles.(D) Time-course NanoSight nanoparticle tracking analysis of NV103 size distribution.

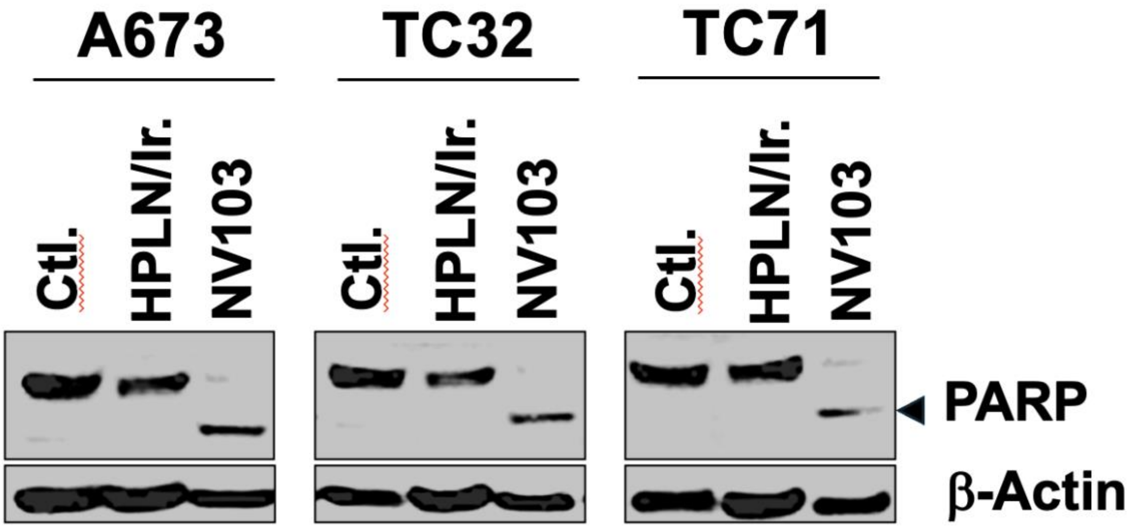

Supplement Figure 3. Induction of Apoptosis by NV103. A673 cells were treated with 5 mM HPLN/Ir or NV103 for two hours. The cells were then washed with fresh media, followed by 18 hours of culture. Lysates from treated cells were assayed for PARP cleavage by Western blotting using antibodies against PARP (arrowhead, cleaved PARP). β-Actin was used as a loading control.

bioRxiv preprint doi: https://doi.org/10.64898/2026.01.06.697855; this version posted January 6, 2026. The copyright holder for this preprint (which was not certified by peer review) is the author/funder, who has granted bioRxiv a license to display the preprint in perpetuity. It is made available under aCC-BY 4.0 International license.

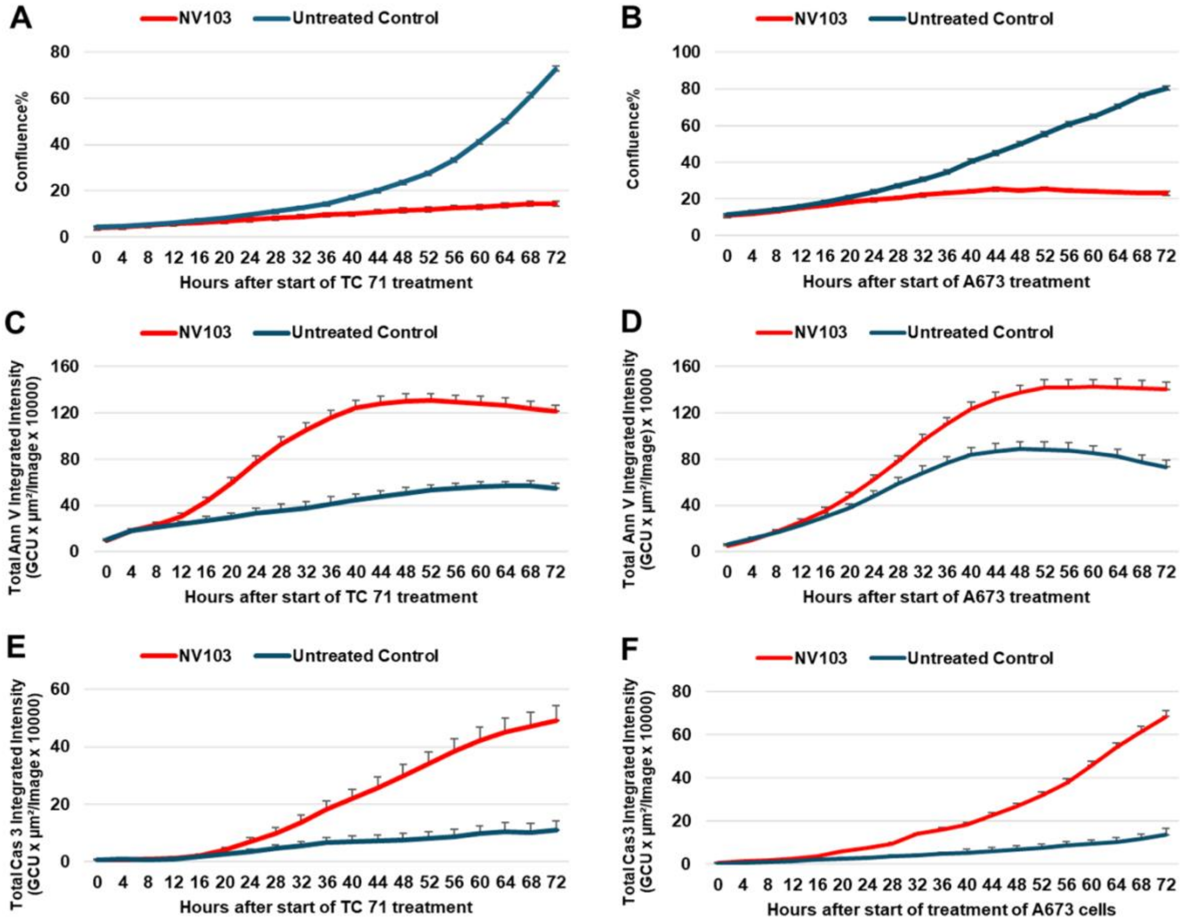

Supplement Figure 4. Onset of irinotecan-induced apoptosis in Ewing sarcoma cell lines following NV103 treatment using Incucyte. Cellular confluence percentage was measured over 72 h in TC71 (A) and A673 (B) Ewing sarcoma cells. Initiation of early apoptosis before 24 h as seen with detection of marker, Annexin V, in TC71 (C) and A673 (D) cells treated with nanoparticles. Late apoptosis was detected after 24 h using the caspase 3 assay in TC71 (E) and A673 (F) cells.

bioRxiv preprint doi: https://doi.org/10.64898/2026.01.06.697855; this version posted January 6, 2026. The copyright holder for this preprint (which was not certified by peer review) is the author/funder, who has granted bioRxiv a license to display the preprint in perpetuity. It is made available under aCC-BY 4.0 International license.

Dose mg/kg Route	Animal ID	Time (hr)	Plasma concentration (ng/mL)[a]	
			Irinotecan	SN-38
Group 1 5 mg/kg IV	22	0.00	BLQ	BLQ
	23	0.00	BLQ	BLQ
	24	0.00	BLQ	BLQ
	1	0.25	30600.00	5350.00
	2	0.25	51000.00	8750.00
	3	0.25	43050.00	12300.00
	4	0.50	63700.00	8113.00
	5	0.50	61550.00	8504.00
	6	0.50	156000.00	5163.00
	7	1.00	46150.00	11114.00
	8	1.00	60750.00	8740.00
	9	1.00	42100.00	9432.00
	10	2.00	34850.00	10743.00
	11	2.00	37150.00	11888.00
	12	2.00	35200.00	8910.00
	13	4.00	29450.00	911.60
	14	4.00	14210.00	1490.00
	15	4.00	28760.00	951.00
	16	8.00	5000.00	974.00
	17	8.00	8180.00	2525.60
	18	8.00	9520.00	1330.80
	19	24.00	420.00	38.40
	20	24.00	310.00	985.60
	21	24.00	980.00	97.20

Supplement Table 1. Individual levels of Irinotecan and SN-38 in plasma following a single intravenous (IV) dose of NV103 at 5 mg/kg in C57BL/6 mice.
BLQ: below limit of quantitation (LOQ=2.5 ng/mL)
Samples were diluted as needed to fall within standard curve

bioRxiv preprint doi: https://doi.org/10.64898/2026.01.06.697855; this version posted January 6, 2026. The copyright holder for this preprint (which was not certified by peer review) is the author/funder, who has granted bioRxiv a license to display the preprint in perpetuity. It is made available under aCC-BY 4.0 International license.

Time (hr)	Irinotecan concentration (ng/mL)		SN-38 concentration (ng/mL)	
	Ave	StdDev	Ave	StdDev
0.00	BLQ	NA	BLQ	NA
0.25	41550.00	10282.39	8800.00	3475.27
0.50	93750.00	53920.80	7260.00	1826.55
1.00	49666.67	9809.73	9762.00	1220.92
2.00	35733.33	1239.29	10513.67	1502.19
4.00	24140.00	8606.55	1117.53	323.17
8.00	7566.67	2321.58	1610.13	812.64
24.00	570.00	359.30	373.73	530.71

Supplement Table 2. Summary of plasma levels of Irinotecan and SN-38 following a single IV dose of 5 mg/kg NV-103 (Group 1) in C57BL/6 mice.
BLQ: below limit of quantitation (LOQ=2.5 ng/mL)
NA: value not available

bioRxiv preprint doi: https://doi.org/10.64898/2026.01.06.697855; this version posted January 6, 2026. The copyright holder for this preprint (which was not certified by peer review) is the author/funder, who has granted bioRxiv a license to display the preprint in perpetuity. It is made available under aCC-BY 4.0 International license.

Dose mg/kg Route	Animal ID	Time (hr)	Plasma concentration (ng/mL)[a]	
			Irinotecan	SN-38
Group 2 50 mg/kg IV	22	0.00	BLQ	BLQ
	23	0.00	BLQ	BLQ
	24	0.00	BLQ	BLQ
	1	0.25	25210.00	3849.00
	2	0.25	28375.00	4366.00
	3	0.25	20140.00	5165.00
	4	0.50	23835.00	3172.00
	5	0.50	28715.00	3166.00
	6	0.50	30485.00	4165.00
	7	1.00	15605.00	1545.00
	8	1.00	18675.00	2706.00
	9	1.00	22405.00	2637.00
	10	2.00	11145.00	2872.00
	11	2.00	7745.00	1599.00
	12	2.00	9080.00	1602.00
	13	4.00	958.00	241.20
	14	4.00	1233.40	315.20
	15	4.00	567.80	288.80
	16	8.00	23.20	50.00
	17	8.00	36.40	50.20
	18	8.00	19.20	23.40
	19	24.00	8.20	9.60
	20	24.00	BLQ	BLQ
	21	24.00	BLQ	BLQ

Supplement Table 3. Individual levels of Irinotecan and SN-38 in plasma following a single IV dose of irinotecan, a standard agent, at 50 mg/kg in C57BL/6 mice.
BLQ: below limit of quantitation (LOQ=2.5 ng/mL)
Samples were diluted as needed to fall within standard curve

bioRxiv preprint doi: https://doi.org/10.64898/2026.01.06.697855; this version posted January 6, 2026. The copyright holder for this preprint (which was not certified by peer review) is the author/funder, who has granted bioRxiv a license to display the preprint in perpetuity. It is made available under aCC-BY 4.0 International license.

Time (hr)	Irinotecan concentration (ng/mL)		SN-38 concentration (ng/mL)	
	Ave	StdDev	Ave	StdDev
0.00	BLQ	NA	BLQ	NA
0.25	24575.00	4154.06	4460.00	663.02
0.50	27678.33	3444.07	3501.00	575.05
1.00	18895.00	3405.33	2296.00	651.30
2.00	9323.33	1713.01	2024.33	734.10
4.00	919.73	334.45	281.73	37.50
8.00	26.27	9.00	41.20	15.42
24.00	8.20	NA	9.60	NA

Supplement Table 4. Summary of plasma levels of Irinotecan and SN-38 following a single intravenous dose of irinotecan, a standard agent (Group 2), at 50 mg/kg in C57BL/6 mice.
BLQ: below limit of quantitation (LOQ=2.5 ng/mL)
NA: value not available
